# Implementing a personalized pharmaceutical plan in kidney or liver transplant patients: study protocol for a stepped-wedge cluster randomized trial (GRePH)

**DOI:** 10.1186/s13063-021-05749-w

**Published:** 2021-11-08

**Authors:** Xavier Pourrat, Elise Berthy, Antoine Dupuis, Louise Barbier, Matthias Buchler, Leslie Grammatico Guillon, Fanny Monmousseau, Eric Ruspini, Ephrem Salamé, Solène Brunet Houdard, Bruno Giraudeau

**Affiliations:** 1 Pharmacy Department, Pharm D, Tours University Hospital, 2 boulevard Tonnelle, 37044, 09 Tours Cedex, France; 2 Biology-Pharmacy-Public Health Department, University Hospital of Poitiers, 2 rue de la 9 Milétrie, 86021 Poitiers Cedex, France; 3 Digestive Surgery and Liver Transplantation, Tours University Hospital, Tours, France; 4 Nephrology Department, Tours University Hospital, 2 boulevard Tonnelle, 37044, 09 Tours Cedex, France; 5 Department of Medical Information, Epidemiology and Medical Economy, Tours University Hospital, Tours, France; 6 INSERM U966, University of Tours, Tours, France; 7 Health-economic Evaluation Unit, University Hospital of Tours, Tours, France; 8 EA 7505 Education, Ethics, Health, University of Tours, Tours, France; 9 Regional Union of Healthcare Professionals Pharmacists of the Greater East of France, 4 rue Piroux, Nancy, France; 10 Université de Tours, Université de Nantes, INSERM, SPHERE U1246, Tours, France; 11 INSERM CIC1415, CHRU de Tours, Tours, France

**Keywords:** Personalized pharmaceutical plan, Therapeutic adherence, Immunosuppressive drugs, Clinical pharmacy, Community-hospital link, Transplantation, Medico-economic study, Stepped-wedge cluster randomized trial

## Abstract

**Background:**

Nowadays, the main challenge of transplantation is the improvement of long-term care, aiming at reducing treatment-related complications and at decreasing rejection rates. Patients’ adherence to both treatment and hygienic-dietary measures is mandatory to achieve these objectives. Adherence to immunosuppressive drugs is estimated to be only 70%. We hypothesized that the implementation of a personalized pharmaceutical plan (PPP) would increase adherence and therefore graft survival.

**Methods/design:**

This study is a stepped-wedge cluster randomized trial with transplantation units defining clusters. Twelve clusters from 10 university hospitals were recruited. All centres started on the same day in the control phase. Every 7 weeks, one centre will switch to the intervention phase and remain there until the end of inclusions. We plan to recruit 1716 kidney and/or liver transplant patients. The intervention phase consists in setting up the PPP: development of the patient’s hospital and community pharmaceutical follow-up. In the hospital, the pharmacist will carry out drug reconciliation upon admission, daily pharmaceutical follow-up of prescriptions and pharmaceutical interviews with the patient in order to explain the modalities of taking immunosuppressive drugs and hygienic-dietary measures. After hospitalization, during the post-transplantation year, pharmaceutical meetings will take place, prior to medical consultations in order to check the patient’s understanding of the prescription, his adherence, to remind them of hygienic-dietary measures and to look for adverse effects. The hospital pharmacist will also be in charge of establishing a close link with the community pharmacist (CP) and general practitioner, especially providing discharge medication reconciliation, an e-learning and a checklist. Moreover, prior to each pharmaceutical consultation, the hospital pharmacist will contact the CP to discuss patient adherence.

The primary outcome is adherence to immunosuppressive treatments 1 year post-transplantation assessed by using the BAASIS questionnaire and the health insurance data from the national health data system. A medico-economic study will measure the efficiency of this plan.

**Discussion:**

GRePH aims to increase adherence of liver and/or kidney transplant patients to their immunosuppressive therapies in order to reduce transplant rejections. To this end, a new clinical pharmacy model, the PPP, will be set up in 10 university hospitals.

**Trial registration:**

ClinicalTrials.gov NCT04295928. Registered on 5 March 2020

**Supplementary Information:**

The online version contains supplementary material available at 10.1186/s13063-021-05749-w.

## Background

In 2019, 6107 solid organ transplants were performed in France, with an annual increase of 1.6% and of 10% over 5 years. At the end of 2016, 22,617 patients were on the transplant waiting list. The two most concerned organs by transplantation were the kidney and liver with respectively 3641 and 1355 procedures performed [1]. Kidney transplantation (KT) is indicated for end-stage renal failure, especially in dialysis patients. Indications for liver transplantation (LT) are primarily acute or chronic hepatic failure and liver cancers. Kidney and liver transplantations allow patients to live almost normally, though they must take immunosuppressive drugs for the rest of their lives. In the last few years, progress has been made both in surgery and in immediate post-transplantation management. Therefore, the challenges of transplantation now lie in long-term transplant patient management, i.e. in the prevention of transplant organ rejection and of immunosuppressive drug side effects (cardiovascular diseases, cancers, infections). In fact, in 2017, graft survival rates at 5 and 10 years were respectively only 79 and 62% for KT and 69 and 58% for LT [2]. Moreover, the scarcity of grafts, in France, makes it necessary to maximize graft survival in order to reduce the rate of retransplantation [3]. This prevention of organ rejection requires optimum patient adherence to both drug treatment and dietary measures. Moreover, compliance with hygienic-dietary measures could partially limit adverse effects and also contribute to increase adherence.

In the literature, adherence of anti-rejection drugs is between 45 and 85% [4]. This variability could be explained by non-standardized evaluation methods from one study to another and to the absence of multi-centre evaluations [5]. In kidney transplant patients, non-adherence, in combination with other factors, is estimated to be responsible for 36% of rejections [6]. Among the risk factors for non-adherence, the lack of therapeutic education programme and the time to transplantation (with the spacing of consultations at the hospital) have been identified: over time, patients tend to be less adherent to their treatment.

We hypothesize that the combination of pharmaceutical follow-up during hospitalization and a better communication between health professionals is capital to patient adherence and therefore to graft and patient survival.

In 2017, the French Society of Clinical Pharmacy (SFPC) established a new model of clinical pharmacy [7]. This latter is based on the implementation of a personalized pharmaceutical plan (PPP) corresponding to management adapted to the patient’s pathway by proposing the best possible medication history, pharmaceutical interviews, pharmaceutical outpatient consultations and therapeutic education. These actions must be carried out both in hospital and in community pharmacy.

Post-transplantation follow-up is carried out by the transplant centre and, in some cases, according to local constraints, alternating by other centres closer to the patient’s home, but often quite far from the transplant team. There is little involvement of primary health care providers in the management of transplant patients. This is why the pharmacist, in collaboration with the transplant team, could be a relay to the patient ensuring continuity, repetition of messages and follow-up as soon as the post-transplantation consultations spread out. A reinforced community-hospital link between pharmaceutical professionals would help ensure continuity of care and optimized follow-up. Several studies have been conducted to improve patient adherence to treatment. With two exceptions, however, these studies remain monocentric and essentially concern only kidney transplant patients [8].

This protocol describes the GRePH study which aims to evaluate the impact of a PPP with usual practice on therapeutic adherence for immunosuppressive drugs in kidney or liver transplant patients who undergone transplantation in 10 French university hospitals (12 transplantation units).

## Methods/design

### Study design and randomization procedure

This study will be a stepped-wedge cluster randomized trial. Clusters will be 12 transplantation units. In cases where a hospital has both a kidney and liver transplant unit, these units will correspond to two clusters. However, if both kidney and liver transplant patients are managed within the same transplant unit, this latter unit will be considered as a single cluster. In a stepped-wedge trial, all centres start the study on the same day in the control phase. Then, one after the other, at regular time intervals, each cluster will switch to the intervention phase and remain there until the end of inclusions. During the control phase, the practices of the different centres will not be modified. During the intervention phase, the PPP will be deployed.

**Fig. 1:**
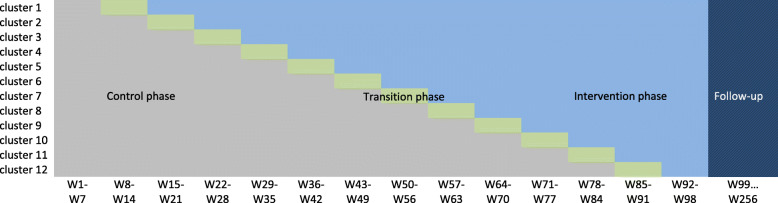
Schematic representation of the stepped-wedge study

In order to gain power, there will be as many sequences (and therefore switch dates) as clusters, meaning that only one cluster at a time will switch from the control phase to the experimental phase. A 7-week transition period will take place at the beginning of all intervention phases, such that the intervention can be rolled out under optimal conditions. The clusters will all be randomized at the beginning of the study and cluster gatekeepers informed of the randomization result. Because some centres had to recruit staff to carry out the intervention phase, communication of the randomization result is mandatory. Randomization will be managed by the biostatistician of the study at the Clinical Investigation Centre (CIC) INSERM 1415. The patient recruitment period will last 23 months with a 3-year follow-up (Fig. 1).

### Clusters and participants

In total, this study will bring together 12 clusters (5 liver clusters, 6 kidney clusters and one mixed cluster) representing 10 French university hospitals (Brest, Marseille, Montpellier, Nantes, Poitiers, Strasbourg, Reims, Rennes, Toulouse and Tours).

Inclusion criteria are as follows: be 18 years old or older; have given their free, informed and express consent; speak French; live in France and have no plan to move for the duration of the study; declare going to the same community pharmacy each time (in France, 81% of French people declare themselves loyal to their community pharmacy [9]); and be health-insured.

Protected patients (safeguard of justice, curatorship or tutorship), already transplanted (whatever organ) or having benefited from a double transplantation (kidney/heart, kidney/liver with a centre not participating in the study or liver/heart) will be excluded.

### Recruitment and inclusion

Patients will be recruited postoperatively as soon as they will be able to understand the purpose of the research. Provided consent is given, all transplant patients meeting the inclusion criteria will be included in the study. The investigator of the centre will ensure the information and collection of the patient’s consent.

Patients are expected to be loyal to a community pharmacy. Consequently, any pharmacy may be involved in this present study. All community pharmacists (CP) will be informed of the study in three ways: a professional journal supported by the pharmacist union, a professional journal supported by the national council of the order of pharmacists and a letter sent by the study scientific committee distributed by wholesale drug distributors. CP having at least one patient included in this study will be informed of the study and the inclusion of their patient by hospital pharmacists. They will be given explanations on the study, how to use the tools and their key role.

### Sample size calculation

Currently, non-adherence to immunosuppressive treatment is approximately 30% [4]. The objective of the intervention is to reduce this percentage by one-third, i.e. to 20%. Considering a stepped-wedge design with 12 sequences (one cluster per sequence) of fourteen 7-week periods (one of which is a transition period), with a mean number of 11 patients recruited per cluster-period, this provides a 90% power to the study, taking into account an intraclass correlation coefficient of 0.01. Consequently, the total number of patients needed is 1716 of whom 132 are expected to be included in a transition period. Sample size calculation has been done using the shiny cluster randomized calculator [10, 11].

According to the French regulatory Agency’s 2017 data, the 12 recruited clusters could in turn recruit 2461 patients over 23 months. Considering that 25% of transplanted patients will have at least one exclusion criterion, 1846 patients will be included over 23 months.

### Control and intervention phase

**Fig. 2:**
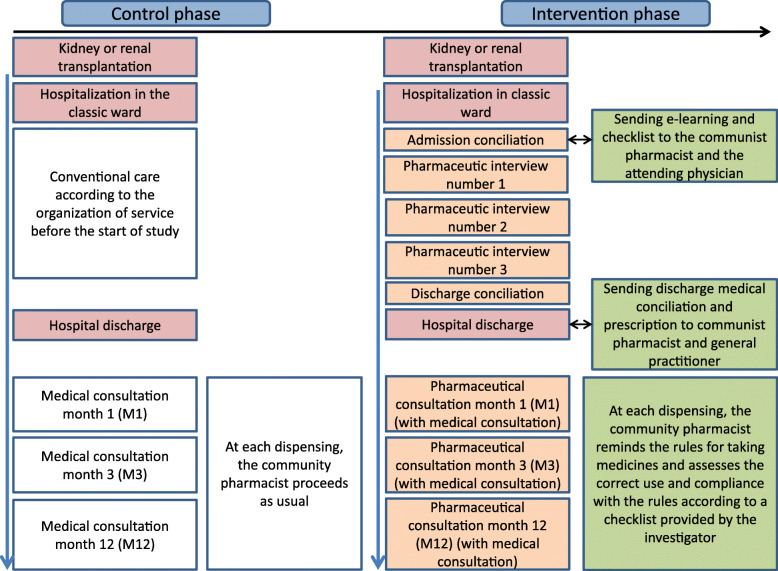
Diagram showing the course of the study

The intervention flowchart is outlined in Fig. 2. During the control phase, patients included will be managed according to usual care. If educational and/or clinical pharmacy activities already exist in some clusters, they will be maintained with the collection of this information.

During the intervention phase, the hospital pharmacist will be in charge of admission and discharge medication reconciliation, 3 pharmaceutical interviews, therapeutic follow-up and communication with the CP. Moreover, he/she will conduct 3 pharmaceutical consultations with the outpatient 1 month (M1), 3 months (M3) and 12 months (M12) after transplantation. To standardize this intervention over the various hospitals, hospital pharmacists will receive a 2-day training in medication reconciliation procedures, pharmaceutical interviews and consultations and therapeutic follow-up by an experienced clinical pharmacist who is accredited by the SFPC. Investigators (i.e. hospital pharmacist) from each centre will be trained at the sponsor centre (Tours University Hospital). Support documents (e-learning, support sheet for conducting pharmaceutical interviews, pharmaceutical consultations, drug reconciliations, standard letters to the attending physician, immunosuppressive drugs tryptic, checklist for CP, etc.) will be given to the trained centre. Each document will have been previously adapted to the current practices of the trained centre and validated by the latter. This training will take place in the 7 weeks preceding the intervention phase.

During hospitalization, the 3 pharmaceutical interviews will focus on anti-rejection drugs, adjuvant treatments to transplantation (anti-infectious chemoprophylaxis, antiviral, treatment of complications of immunosuppressive drugs: diabetes, hypertension, etc.) and hygienic-dietary measures. The first meeting allows the patient to be provided with a panel of simple information. The pharmacist will reformulate the information and explanations on request such that the patient memorizes the message. The second meeting will take the form of open questions. It will aim to assess what the patient has learned and understood. Do they know what time to take their treatments? Do they know what to do about meals? Do they know what precautions to take with regard to the sun? How to carry heavy loads? If necessary, the pharmacist will re-explain all these notions. The pharmacist can repeat this interview number 2 until the patient understands, memorizes, integrates and appropriates the information. In case of major difficulties or for non-autonomous patients, interviews may be conducted with a caregiver (by phone if needed). The third interview will take place on the day of discharge (or the day before if the discharge is scheduled), after a brief reminder of the instructions/information, an intake plan and a pill box for a nychthemeron will be issued to the patient. For each drug, the patient will have to explain in their own words its goal and moment to be taken relative to the time, other medications and/or meals. A report of each meeting will be placed in the patient’s file. These summaries will be available to the medical team in order to prepare the patient’s discharge: is the patient able to manage their treatment at home alone? In addition, they will be sent to the CP with the discharge reconciliation and prescription, thus strengthening the community-hospital link. The CP will thus be aware of the points of vigilance to be discussed with the patient. Discharge medication reconciliation and prescription will also be sent to the patient’s general practitioner (GP). These will specify the treatments initiated, modified or stopped following transplantation. Moreover, an e-learning on transplantation, the objectives to be achieved and patient management will be sent to the CP and GP. This e-learning will present a quiz for the various topics addressed, allowing the professional to check their knowledge. This e-learning will be personalized for each cluster to adapt to local specificities. Finally, a checklist with all information to be specified at the time of each dispensation will be sent to the CP by the hospital pharmacist.

At the time of the M1, M3 and M12 medical consultations, the hospital pharmacist will meet with the patient again. To further involve the patient, a booklet will be issued upon discharge from the hospital in order to inform them of the anticipated dates for the pharmaceutical consultations. For patients hospitalized at M1, M3 or M12, consultations will be staggered over time: they will be performed at the time of the first medical consultation following the patient’s discharge. The hospital pharmacist will carry out a drug assessment, draw up a list of undesirable effects, ensure appropriate drug intake, collect any problems or questions reported by the patient and will ensure the persistence of the educational messages given during the pharmaceutical hospitalization interviews. Also, prior to the consultation, he/she will contact the CP to ensure that this latter is in possession of the medication. A summary of this exchange will be written in the patient’s medical file and presented to the physician immediately prior to the consultation (Additional file 2).

Due to the nature of the assessed intervention, blinding will not be possible for patients and the healthcare team.

### Outcomes

#### Primary outcome measure

The primary outcome is adherence to immunosuppressive treatments 1 year after transplantation (M12). To assess therapeutic adherence (compliance and persistence), a combination of two methods is recommended [12]. Accordingly, we will use the BAASIS (Basel Assessment of Adherence to Immunosuppressive Medication Scale) questionnaire and the health insurance data from the national health data system (SNDS). The BAASIS questionnaire [13] will be used 1 year after the transplantation (M12) (Additional file 3). This is a recognized questionnaire for assessing adherence to immunosuppressive therapy. It includes 4 questions to determine whether, in the last 4 weeks, the patient forgot to take, did not respect the taking schedule (to the nearest 2 h) or changed the dosage of their immunosuppressive treatment. A patient answering “yes” to one of the 4 questions will be treated as a non-adherent.

Using the SNDS health insurance data, we will verify that over the year following the transplantation, 100% of the days are covered by immunosuppressive drug possession by counting the number of tablets dispensed by the CP during this period (for hospitalization days, therapeutic adherence will be estimated at 100%). The SNDS health insurance data are described in Additional file 4.

To be considered as adherent, a patient must be assessed as an adherent on both criteria.

#### Secondary outcome measures

A series of secondary clinical outcomes will be assessed at M12: adherence to all chronic disease drugs assessed with the EvalOBS scale [14], knowledge of hygienic-dietary measures and methods of taking immunosuppressive drugs assessed by a 9-item questionnaire [15] specially created for the study (Additional file 5), occurrence of adverse effects (search for 6 known side effects: hypertension, diabetes, kidney failure, bacterial, viral or parasitic infections) and graft status (graft survival, resumption of dialysis for renal transplant patient, new transplantation, patient death, acute rejection episodes). Via the EvalObs visual scale, the patient has to answer the question “how did you take your treatment in the last month”. For this, he is given a graduated scale from 1 to 17 (1 = I did not take any medication; 17 = I took all the tablets). Below 14, adherence to treatment is considered unsatisfactory.

Patients, practitioners and CP satisfaction will also be assessed thanks to a 5-level Likert scale.

Three years after transplantation (M36), graft status will be reassessed. Moreover, at M36, the risk of potential rejection will also be evaluated thanks to the study of the coefficient of variation (CV) of calcineurin inhibitor dosage. A patient with a CV greater than 30% will be considered to be at higher risk of rejection [16].

Finally, for renal transplant patients, the presence of anti-HLA antibodies will be tested at M12 and M36.

This study also includes a number of secondary health-economic objectives. The efficiency of PPP implementation will be evaluated over 1 year, performing a cost-utility analysis and two cost-effectiveness analyses from the French Healthcare Insurance perspective. The cost-utility endpoint will be the cost per QALY gained at 12 months. Quality of life data will be collected using the validated French EQ5D-5L survey, administered at 0, 1, 3, 6, 9 and 12 months’ follow-up. These questionnaires will preferably be collected face-to-face during patient visits to the hospital for medical follow-up. Otherwise, they may be collected by phone. The cost-effectiveness endpoints will be the cost per additional adherent patient and the cost per additional first functional graft in living patients at 12 months. Hospital and ambulatory costs, transport expenses and sickness allowances will be valued over a 1-year period. These consumed resources will be collected by specific retrieval from the SNDS. The cost of the PPP will be obtained through a microcosting procedure. For this, the hospital pharmacist will have to time the interviews and consultations.

### Funding source and regulatory aspects

This study is funded by the French Ministry of Health (PREPS number 2018-0486). The Tours University Hospital is the sponsor and is in charge of all administrative measures. The ethics committee (CPP Ile de France III – Hospital Tarnier - Cochin) has approved the study for all centres (reference Am8642-1-3728). The French committee for data handling (CNIL) approved the study (number 920070 dated 29/07/2020). This trial was registered with ClinicalTrials.gov (number: NCT04295928 on March 5, 2020, Establishment of a Personalized Pharmaceutical Plan in Renal or Hepatic Transplant Patients). This protocol was drafted as per the guidelines of the SPIRIT checklist (Additional file 1).

In the event that the sponsor wishes to amend the protocol, a request for a substantive amendment will be drawn up by the sponsor and sent to the CPP for approval. Once the amendment has been approved by the CPP, it will be sent to all the investigators and can be implemented. The protocol will also be updated on ClinicalTrials.gov. At last, any deviations from the protocol will be fully documented using a breach report form.

### Data management

Health professionals with direct access will take all necessary precautions to ensure the confidentiality of information, particularly with regard to their identity and the results obtained. These persons, in the same way as the investigators themselves, are subject to professional secrecy.

For each patient who agrees to participate in the study, the investigator is provided with a follow-up notebook in which he records all the clinical and quality of life information required by the protocol. The investigator is responsible for the accuracy, quality and relevance of data entered. Each notebook is anonymised: data is coded with the first letter of the patient’s surname and first name and a number specific to the research (N1) corresponding to the order of inclusion of the patients.

A copy of these anonymised notebooks will be sent to the coordinating centre by secure mail. In the coordinating centre, a clinical research technician will enter data into the e-CRF.

Access to SNDS data requires patient identification data. After a favourable opinion from the CNIL, each investigating centre will transmit a table of identifying data to a trusted third party (independent of the coordinating investigator, the sponsor and the investigating centres) by a secure e-mail. This table will not contain any medical, pharmaceutical or paramedical data and will comply with a strict format standard. It will contain the patient code in the study (N1), the surname, the first name, the date of birth, the place of birth (postcode + town + country) and the sex. These data will make it possible to reconstitute the beneficiary’s SNDS identifier, used for extractions from the SNDS database. A new anonymous number (N2) will be created for each patient and the SNDS data will be transmitted by this new anonymous number to a secure project space. The trusted third party will send the patient number (N1)—anonymous number (N2) equivalence table to the Health-Economic Evaluation Unit (UEME) of the University Hospital of Tours. Then, the UEME will make the link between the clinical data collected specifically for the study and the SNDS data. The SNDS data will remain strictly on this project space, and no extraction will be performed.

### Analysis

Data will be analysed according to a pre-established statistical analysis plan that will be finalized before locking the database. The results of the study will be reported in accordance with the guidelines of the CONSORT Statement extension for the stepped-wedge cluster randomized trials [9]. In accordance with act no. 2002-303 of 4 March 2002, if they request it, the participants will be informed of the results of the study.

Cluster characteristics will be reported using descriptive statistics as will be patient characteristics, which will be reported per arm.

The primary endpoint is binary: patients adhering to their immunosuppressive treatment or not (assessed using the BAASIS questionnaire and health insurance data 1 year after transplantation). It will be analysed using a mixed logistic regression model taking into account a period effect and a cluster random effect. It will also be analysed using a mixed model with an identity link function (still taking into account both time and clustering), thus allowing to estimate a risk difference. All included patients will be taken into account in the main analysis and considered in the group (control or experimental) corresponding to the period during which they have been included, whatever occurred. In case the BAASIS questionnaire is missing, we will consider health insurance data (SNDS data) only to specify whether a patient is adherent or not. Because health insurance data are routinely collected data for all French people, we do not expect any missing data. No subgroup analyses or adjusted analyses are planned.

Binary secondary endpoints (adhesion, graft survival at 1 and 3 years, appearance of HLA antibodies, presence of episodes of acute rejection) will be analysed using the same approach as that used for the primary endpoint. For continuous secondary criteria (knowledge of the patient, patient satisfaction, coefficient of variation of the dosages), a mixed linear model will be used taking into account a period effect and a cluster random effect.

Data will be analysed by statisticians from the CIC Centre INSERM 1415 using SAS or R software.

The cost-utility and cost-effectiveness analyses will follow the French guidelines for economic evaluation in health care.

After the data management of SNDS data by the EpiDclic Unit, the health-economic analysis will be performed by UEME. It will be performed using the following software: Excel, Stata and R.

An initial analysis will be carried out once all M12 data will be collected. Then, another analysis will be carried out with the data collected 3 years after the transplant.

### Trial Steering Committee

The Trial Steering Committee is comprised of hospital and community pharmacists, liver transplant surgeon and nephrologist, biostatistitians and public health doctors. The study was designed by this scientific committee. This committee, with the help of the coordinating clinical research officer (CRO), is also responsible for obtaining all regulatory approvals. To this end, monthly phone meetings are held to discuss the progress of the project.

Once inclusions are opened, the scientific committee meet regularly to discuss the progress of the inclusions and to respond to any difficulties encountered by the centres. The coordinator also ensures that the planning of the study is respected (respect of switch dates in the intervention phase, training of centres). A monthly newsletter, written by the scientific committee and the coordinating CRO, is sent to all the investigating centres.

## Discussion

GRePH aims to evaluate the impact of a PPP on therapeutic compliance for immunosuppressive drugs in kidney or liver transplant patients and consequently on graft and patient survival. Indeed, according to the literature, only 45 to 85% of patients on immunosuppressive drugs take their immunosuppressive treatments correctly [4]. However, patients’ compliance with their anti-rejection treatments is essential for graft survival. Given the scarcity of transplants [3], it seems essential to improve patients’ compliance with their treatments.

As a lack of therapeutic education has been identified as a factor in poor compliance, this PPP aims to reinforce patients’ knowledge of their treatments. Better informed about the importance of anti-rejection drugs, how to take them and the precautions to take to avoid possible side effects, we believe that patients will be more vigilant.

Similarly, this PPP aims to involve CPs and GPs in the follow-up of transplant patients. Indeed, even today, activities carried out in the hospital and in private practices remain separate. However, once out of the hospital, due to their proximity, CPs and GPs are often patients’ primary contacts. However, due to the small number of transplant patients, CPs are often unfamiliar with immunosuppressive drugs. This PPP aims to provide them with ample information on the management of transplant patients in private practice. Thus, if necessary, CPs will be able to remind transplant patients of the procedures for taking anti-rejection drugs, the importance of good compliance with these treatments and the importance of following lifestyle and dietary recommendations. These reminders may possibly compensate for the lower level of compliance with treatment observed some time after a transplant.

Through this PPP, we intend to improve the therapeutic education of patients and also strengthen the private practice-hospital link in the aim of increasing patients’ adherence to their immunosuppressive treatments by 70 to 80%.

Thanks to GRePH, this innovative PPP will be implemented in 10 French university hospitals. The stepped-wedge design was chosen in order to maintain the PPP once the study is completed. Indeed, thanks to this scheme, at the end of the inclusions, all the centres will still be in the intervention phase, an important criterion that can strengthen the participation of centres in the study. Moreover, a stepped-wedge design avoids group contamination: at a given time, within the service, all patients will participate either in the control phase or in the intervention phase.

Finally, a medico-economic study will evaluate the efficiency of PPP. GRePH will be the first study to evaluate the benefits of implementing PPP in transplant patients. If its effectiveness is demonstrated, this process could be generalized to all transplant patients. It could even be applied to other pathologies. Last but not least, through GRePH, close links between hospital and community professionals will be established, which could reduce rehospitalization in the long term.

## Trial status

All centres started the study in the control phase on 7 October 2020. The first centre has entered the intervention phase on 11 January 2021. Since then, 5 other centres have joined it. Another centre is in the transition phase and carrying out its 2-day training via the sponsor. At this time, 526 patients have been included. Protocol version 2.3 (8 July 2020). The theoretical end date for inclusion is 28 August 2022.

## Supplementary Information

**Additional file 1.** Spirit checklist.

**Additional file 2.** Support for pharmaceutical consultations.

**Additional file 3.** BAASIS questionnaire [13].

**Additional file 4.** SNDS data.

**Additional file 5.** Knowledge of hygienic and dietary measures questionnaire.

## Data Availability

Data will be collected and managed by the Tours University Hospital. Conditions for transfer of all or part of the anonymous database will be decided by the study sponsor and will be the subject of a written contract. This trial does not involve collecting biological specimens for storage.

## Abbreviations

BAASIS: Basel Assessment of Adherence to Immunosuppressive Medication Scale
CIC: Clinical Investigation Centre
CNIL: French committee for data handling
CP: Community pharmacist
CRO: Clinical research officer
CV: Coefficient of variation
EpiDcliC: Epidemiology Unit of the Centre Loire Valley Region
GP: General practitioner
KT: Kidney transplantation
LT: Liver transplantation
M1: One month after transplantation
M3: Three months after transplantation
M12: Twelve months after transplantation
M36: Thirty-six months after transplantation
PPP: Personalized pharmaceutical plan
SFPC: French Society of Clinical Pharmacy
SNDS: French national health data system
UEME: Health-Economic Evaluation Unit
