# Correction: Implementing a personalized pharmaceutical plan in kidney or liver transplant patients: study protocol for a stepped-wedge cluster randomized trial (GRePH)

**DOI:** 10.1186/s13063-022-06381-y

**Published:** 2022-05-24

**Authors:** Xavier Pourrat, Elise Berthy, Antoine Dupuis, Louise Barbier, Matthias Buchler, Leslie Grammatico Guillon, Fanny Monmousseau, Eric Ruspini, Ephrem Salamé, Solène Brunet Houdard, Bruno Giraudeau

**Affiliations:** 1 Pharmacy Department, Pharm D, Tours University Hospital, 2 boulevard Tonnelle, 37044, 09 Tours Cedex, France; 2 Biology-Pharmacy-Public Health Department, University Hospital of Poitiers, 2 rue de la 9 Milétrie, 86021 Poitiers Cedex, France; 3 Digestive Surgery and Liver Transplantation, Tours University Hospital, Tours, France; 4 Nephrology Department, Tours University Hospital, 2 boulevard Tonnelle, 37044, 09 Tours Cedex, France; 5 Department of Medical Information, Epidemiology and Medical Economy, Tours University Hospital, Tours, France; 6 INSERM U966, University of Tours, Tours, France; 7 Health-economic Evaluation Unit, University Hospital of Tours, Tours, France; 8 EA 7505 Education, Ethics, Health, University of Tours, Tours, France; 9 Regional Union of Healthcare Professionals Pharmacists of the Greater East of France, 4 rue Piroux, Nancy, France; 10 Université de Tours, Université de Nantes, INSERM, SPHERE U1246, Tours, France; 11 INSERM CIC1415, CHRU de Tours, Tours, France

**Correction to: Trials 22, 782 (2021)**

**https://doi.org/10.1186/s13063-021-05749-w**

Following the publication of the original article [1], we were notified that BAASIS scale presented in Additional file 3 should be been removed.

The original article has been corrected.
