# Supplementary figures and images for: Implementing a personalized pharmaceutical plan in kidney or liver transplant patients: study protocol for a stepped-wedge cluster randomized trial (GRePH)
Source: Trials. 2021 Nov 8;22:782. doi: 10.1186/s13063-021-05749-w (PMC8573912; doi:10.1186/s13063-021-05749-w)

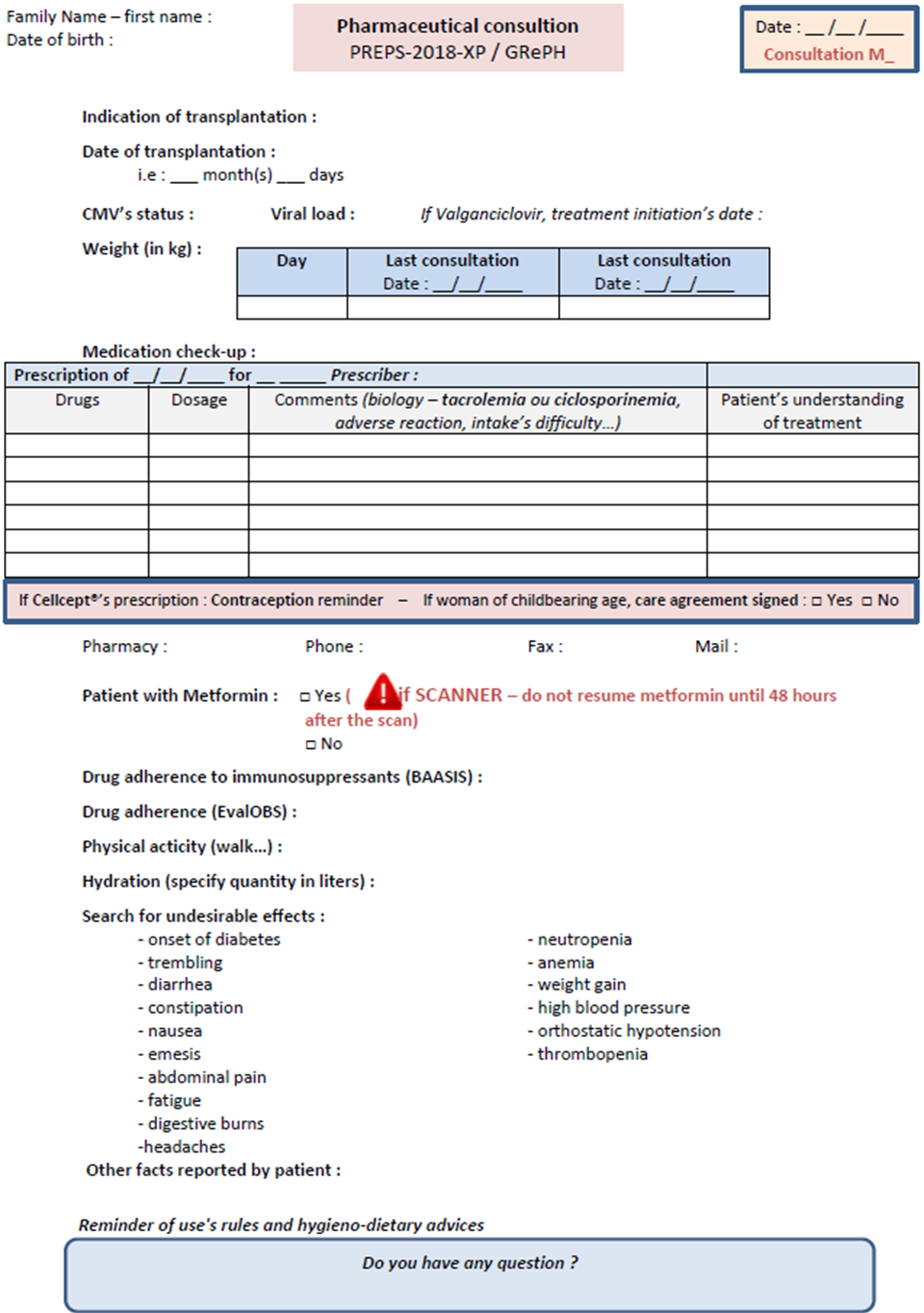


**Pharmaceutical consultation**

**PREPS-2018-XP / GRePH**

Supplement: Supplementary file 2 — Additional file 2. Support for pharmaceutical consultations. [file 13063_2021_5749_MOESM2_ESM.docx]
