# Supplementary material for: Implementing a personalized pharmaceutical plan in kidney or liver transplant patients: study protocol for a stepped-wedge cluster randomized trial (GRePH)
Source: Trials. 2021 Nov 8;22:782. doi: 10.1186/s13063-021-05749-w (PMC8573912; doi:10.1186/s13063-021-05749-w)
Supplement: Supplementary file 3 — Additional file 3. BAASIS questionnaire [13]. [file 13063_2021_5749_MOESM3_ESM.pdf]

BAASIS® Questionnaire

University of Basel, Leuven-Basel Research Group, Institute of Nursing Science, Department Public Health, University of Basel, Switzerland.

1A. Do you remember missing a dose of any of your immunosuppressive drugs in the last four weeks? □ YES □ NO

If yes, could you tell me how many times this has happened to you in the last four weeks? □ Once □ Twice □ Three times □ Four times □ More than four times

1B. Do you remember missing at least two successive doses of your immunosuppressive medication in the last four weeks? □ YES □ NO

If yes, could you tell me how many times this has happened to you in the last four weeks? □ Once □ Twice □ Three times □ Four times □ More than four times

2. Your immunosuppressive drugs must be taken at specific times, as recommended by your transplant team. In the last four weeks, do you remember taking your immunosuppressive drugs more than two hours before or after the prescribed time? □ YES □ NO

If yes, could you tell me how often this has happened to you in the last four weeks? □ Once □ Two to 3 times □ Four to 5 times □ Every 2 or 3 days □ Almost every day

3. Have you ever changed the number of immunosuppressive drugs prescribed (e.g. by taking more or less tablets) in the last four weeks without informing your doctor? □ YES □ NO

4. Have you stopped taking your immunosuppressive medication completely in the past year without informing your doctor? □ YES □ NO

5. Finally, we would like to ask you to rate the way you have taken your immunosuppressive medication in the last four weeks.

Using the rating scale below, could you please indicate to us, by means of a percentage, the extent to which you feel you have taken your immunosuppressive medication exactly as prescribed (please take into account the actual intake and time of day) over the last four weeks?

For example:

0% = I have never taken my medication as prescribed

50% = I took my medication as prescribed half the time

100% = I always took my medication as prescribed

|___|___|___|
