# Supplementary material for: Implementing a personalized pharmaceutical plan in kidney or liver transplant patients: study protocol for a stepped-wedge cluster randomized trial (GRePH)
Source: Trials. 2021 Nov 8;22:782. doi: 10.1186/s13063-021-05749-w (PMC8573912; doi:10.1186/s13063-021-05749-w)
Supplement: Supplementary file 4 — Additional file 4. SNDS data. [file 13063_2021_5749_MOESM4_ESM.pdf]

SNDS Data

Unique in Europe, and even in the world, the National Health Data System (SNDS) is a data warehouse bringing together data from hospital billings and primary care professionals.

For each treatment consumed, the SNDS contains the type of treatment (consultation with a general practitioner, physiotherapy, blood tests, hospitalization, etc.), its price, and the amount reimbursed by the French health insurance system and supplementary health insurance. Deaths (dates and causes) are also recorded in the SNDS. The SNDS also contains the reimbursement rate of patients, and in particular, the "long-term condition" status granted to transplant patients, which allows them to have their care related to the transplant covered at 100%.

The SNDS is exhaustive for the entire French population, which makes the data on reimbursed care reliable. However, with respect to drugs, the SNDS reflects the quantity of drugs billed to the health insurance system, not the quantity of drugs taken by the patient. This is why the primary endpoint of this study is a mixed endpoint based on drug consumption in the SNDS and assessment of compliance by the BAASIS questionnaire.

It is the cost of what is purchased that is relevant for the real-life medico-economic evaluation.

The purpose of the SNDS is to make these data available for studies, research or evaluation of public interest and to contribute to one of the following purposes:

- information on health ;
- the implementation of health policies;
- knowledge of health expenditure;
- informing professionals and institutions about their activities;

- innovation in the fields of health and medico-social care;

- surveillance, monitoring and health security.
