# Supplementary material for: Implementing a personalized pharmaceutical plan in kidney or liver transplant patients: study protocol for a stepped-wedge cluster randomized trial (GRePH)
Source: Trials. 2021 Nov 8;22:782. doi: 10.1186/s13063-021-05749-w (PMC8573912; doi:10.1186/s13063-021-05749-w)
Supplement: Supplementary file 5 — Additional file 5. Knowledge of hygienic and dietary measures questionnaire. [file 13063_2021_5749_MOESM5_ESM.pdf]

Knowledge of hygienic and dietary measures' questionnaire

Preamble: Can you list below the name(s) of your immunosuppressant or anti-rejection medication(s) and the dose to be taken each day?

……………………………………………………………………………………………

1- At what time do you take your anti-rejection medication?

|___|___| hour(s) |___|___| minute(s)

2-Which of the following foods are you allowed to consume without restriction?

- Orange juice ☐ YES ☐ NO
- Seafood ☐ YES ☐ NO
- Roast chicken ☐ YES ☐ NO
- Tomato salad ☐ YES ☐ NO
- Grapefruit juice ☐ YES ☐ NO

3-If I have to go for a walk in the summer, I will

- Take a bottle of water to avoid dehydration ☐ YES ☐ NO
- Take a tube of sunscreen that I would apply if the sun is too strong ☐ YES ☐ NO
- Put a hat on my head ☐ YES ☐ NO
- Wear sunscreen before leaving and reapply if the walk lasts several hours ☐ YES ☐ NO
- Prefers to stay home from the sun ☐ YES ☐ NO

4-The day of the anti-rejection blood test, I take this one:

☐ Just before the blood test ☐ Just after the blood test ☐ Two hours before the blood test ☐ Two hours after the blood test ☐ When I can regardless of the time of the blood test

5-I have a fever of 39°,

- I take paracetamol ☐ YES ☐ NO
- I phone the transplant center ☐ YES ☐ NO
- I am taking the antibiotic that the doctor prescribed last time ☐ YES ☐ NO
- I go to the emergency room of the nearest hospital ☐ YES ☐ NO
- I wait until the next day to see if the fever goes down ☐ YES ☐ NO

6-If I forget the time to take my anti-rejection medication(s)

☐ I take it (them) immediately whatever the hour ☐ I take it (them) within a maximum of 4 hours after the theoretical hour ☐ I wait for the next dose whatever the hour and I double the dose ☐ I wait for the next dose whatever the hour and I take the usual dose ☐ I phone the transplant center or ask my pharmacist ☐ I never forget a dose

7-Concerning all my medications

I take only those prescribed to me ☐ YES ☐ NO

When I consult a dentist or a doctor who is not the one who is following me for the transplant I tell him to check that it is compatible with my transplant ☐ YES ☐ NO

Apart from those prescribed I use phytotherapy or aromatherapy ☐ YES ☐ NO

I buy supplements/fortifiers on the internet ☐ YES ☐ NO

When I have a doubt I ask my pharmacist or the doctor following me ☐ YES ☐ NO

8-Sometimes I take a lower dose of anti-rejection drugs because:

I am tired ☐ YES ☐ NO

I don't have enough and I couldn't go to the pharmacy ☐ YES ☐ NO

I have a fever ☐ YES ☐ NO

I have stomach pain ☐ YES ☐ NO

I think these drugs are hurting me ☐ YES ☐ NO

SCORE: number of correct answers: ___ / 9 (8 questions + names of immunosuppressants) i.e. ____ % of correct answers
