# Supplementary material for: Implementing a personalized pharmaceutical plan in kidney or liver transplant patients: study protocol for a stepped-wedge cluster randomized trial (GRePH)
Source: Trials. 2021 Nov 8;22:782. doi: 10.1186/s13063-021-05749-w (PMC8573912; doi:10.1186/s13063-021-05749-w)
Supplement: Supplementary file 1 — Additional file 1. Spirit checklist. [file 13063_2021_5749_MOESM1_ESM.zip › Additional file 1 Spirit ChecklistR1.doc]

SPIRIT 2013 Checklist: Recommended items to address in a clinical trial protocol and related documents*

| Section/item | ItemNo | Description |
| --- | --- | --- |
| **Administrative information** | | |
| Title | 1 | Descriptive title identifying the study design, population, interventions, and, if applicable, trial acronym (page 1, lines 1-3) |
| Trial registration | 2a | Trial identifier and registry name. If not yet registered, name of intended registry (page 13, line 13) |
|  | 2b | All items from the World Health Organization Trial Registration Data Set (All WHO Trial Registration Data requirements are met with the trial’s registration in the ClinicalTrials.gov. Trial registration information is found on page 13, line 13) |
| Protocol version | 3 | Date and version identifier (page 16, line 23) |
| Funding | 4 | Sources and types of financial, material, and other support (page 19, lines 1-4) |
| Roles and responsibilities | 5a | Names, affiliations, and roles of protocol contributors (page 18, lines 7-22 ; page 20, lines 10-21) |
|  | 5b | Name and contact information for the trial sponsor (page 13, lines 8-9) |
|  | 5c | Role of study sponsor and funders, if any, in study design; collection, management, analysis, and interpretation of data; writing of the report; and the decision to submit the report for publication, including whether they will have ultimate authority over any of these activities (pages 5, lines 11-13 ; page 14, lines 13-14 ; page 14, lines 17-19 ; page 18, lines 1-5 ; page 19 ; lines 14-17) |
|  | 5d | Composition, roles, and responsibilities of the coordinating centre, steering committee, endpoint adjudication committee, data management team, and other individuals or groups overseeing the trial, if applicable (see Item 21a for data monitoring committee) (pages 5, lines 11-13 ; page 14, lines 13-14 ; page 14, lines 17-19 ; page 18, lines 1-5 ; page 19 ; lines 14-17) |
| Introduction |  |  |
| Background and rationale | 6a | Description of research question and justification for undertaking the trial, including summary of relevant studies (published and unpublished) examining benefits and harms for each intervention (page 2, lines 18-29 ; page 3, lines 1-24 ; page 4, lines 1-14) |
|  | 6b | Explanation for choice of comparators (page 3, lines 16-18) |
| Objectives | 7 | Specific objectives or hypotheses (page 4, lines 11-14) |
| Trial design | 8 | Description of trial design including type of trial (eg, parallel group, crossover, factorial, single group), allocation ratio, and framework (eg, superiority, equivalence, noninferiority, exploratory) (page 4, lines 18-24 ; page 5, lines 1-17) |
| Methods: Participants, interventions, and outcomes | | |
| Study setting | 9 | Description of study settings (eg, community clinic, academic hospital) and list of countries where data will be collected. Reference to where list of study sites can be obtained (page 6, lines 2-4) |
| Eligibility criteria | 10 | Inclusion and exclusion criteria for participants. If applicable, eligibility criteria for study centres and individuals who will perform the interventions (eg, surgeons, psychotherapists) (page 6, lines 5-11) |
| Interventions | 11a | Interventions for each group with sufficient detail to allow replication, including how and when they will be administered (page 8, lines 1-13 ; page 9, lines 1-24 ; page 10, lines 1-24 ; page 11, lines 1-5) |
|  | 11b | Criteria for discontinuing or modifying allocated interventions for a given trial participant (eg, drug dose change in response to harms, participant request, or improving/worsening disease) (page 9, lines 14-15, 19-22 ; page 10, lines 19-21) |
|  | 11c | Strategies to improve adherence to intervention protocols, and any procedures for monitoring adherence (eg, drug tablet return, laboratory tests) (Not applicable: this is a pharmaceutical monitoring process designed in such a way that the patient does not have to travel just for the study) |
|  | 11d | Relevant concomitant care and interventions that are permitted or prohibited during the trial (page 5, lines 1-2 ; page 8, lines 5-7) |
| Outcomes | 12 | Primary, secondary, and other outcomes, including the specific measurement variable (eg, systolic blood pressure), analysis metric (eg, change from baseline, final value, time to event), method of aggregation (eg, median, proportion), and time point for each outcome. Explanation of the clinical relevance of chosen efficacy and harm outcomes is strongly recommended (page 11, lines 7-24 ; page 12, lines 1-24 ; page 13, lines 1-5) |
| Participant timeline | 13 | Time schedule of enrolment, interventions (including any run-ins and washouts), assessments, and visits for participants. A schematic diagram is highly recommended (see Figure) (page 8, lines 3) |
| Sample size | 14 | Estimated number of participants needed to achieve study objectives and how it was determined, including clinical and statistical assumptions supporting any sample size calculations (page 7, lines 4-14) |
| Recruitment | 15 | Strategies for achieving adequate participant enrolment to reach target sample size (page 7, lines 12-14) |
| **Methods: Assignment of interventions (for controlled trials)** | | |
| Allocation: |  |  |
| Sequence generation | 16a | Method of generating the allocation sequence (eg, computer-generated random numbers), and list of any factors for stratification. To reduce predictability of a random sequence, details of any planned restriction (eg, blocking) should be provided in a separate document that is unavailable to those who enrol participants or assign interventions (page 4, lines 19-24 ; page 5, lines 6-7 ; page 5, lines 8-12 ; page 6, lines 2-4) |
| Allocation concealment mechanism | 16b | Mechanism of implementing the allocation sequence (eg, central telephone; sequentially numbered, opaque, sealed envelopes), describing any steps to conceal the sequence until interventions are assigned (page 5, lines 8-10) |
| Implementation | 16c | Who will generate the allocation sequence, who will enrol participants, and who will assign participants to interventions (page 5, lines 11-12 ; page 6, lines 14-17) |
| Blinding (masking) | 17a | Who will be blinded after assignment to interventions (eg, trial participants, care providers, outcome assessors, data analysts), and how (page 5, lines 9-10 ; page 11, lines 4-5) |
|  | 17b | If blinded, circumstances under which unblinding is permissible, and procedure for revealing a participant’s allocated intervention during the trial (not applicable : the blind is not possible here, a pharmaceutical process with consultations and interviews to be set up for patients in the intervention phase) |
| **Methods: Data collection, management, and analysis** | | |
| Data collection methods | 18a | Plans for assessment and collection of outcome, baseline, and other trial data, including any related processes to promote data quality (eg, duplicate measurements, training of assessors) and a description of study instruments (eg, questionnaires, laboratory tests) along with their reliability and validity, if known. Reference to where data collection forms can be found, if not in the protocol (page 8 ; lines 12-13 ; page 9, lines 1-9 ; page 10, lines 11-15 ; page 11, lines 11-14, 20-24 ; page 12, lines 2-24 ; page 13, lines 1-5) |
|  | 18b | Plans to promote participant retention and complete follow-up, including list of any outcome data to be collected for participants who discontinue or deviate from intervention protocols (page 10, lines 17-19) |
| Data management | 19 | Plans for data entry, coding, security, and storage, including any related processes to promote data quality (eg, double data entry; range checks for data values). Reference to where details of data management procedures can be found, if not in the protocol (Not applicable. Anonymised data will be centralised in a secure eCRF.) |
| Statistical methods | 20a | Statistical methods for analysing primary and secondary outcomes. Reference to where other details of the statistical analysis plan can be found, if not in the protocol (page 13, lines 21-24 ; page 14, lines 1-21) |
|  | 20b | Methods for any additional analyses (eg, subgroup and adjusted analyses) (page 13, lines 21-24 ; page 14, lines 1-21) |
|  | 20c | Definition of analysis population relating to protocol non-adherence (eg, as randomised analysis), and any statistical methods to handle missing data (eg, multiple imputation) (Not applicable, solutions are put in place so that there is no missing data. In addition, the patient follow-up notebook is very simple in order to mention that the data are indispensable) |
| **Methods: Monitoring** | | |
| Data monitoring | 21a | Composition of data monitoring committee (DMC); summary of its role and reporting structure; statement of whether it is independent from the sponsor and competing interests; and reference to where further details about its charter can be found, if not in the protocol. Alternatively, an explanation of why a DMC is not needed (Not applicable. This is the implementation of a process with data collection only at M12. There is therefore no need to have a data monitoring committee) |
|  | 21b | Description of any interim analyses and stopping guidelines, including who will have access to these interim results and make the final decision to terminate the trial (page 14, lines 20-21) |
| Harms | 22 | Plans for collecting, assessing, reporting, and managing solicited and spontaneously reported adverse events and other unintended effects of trial interventions or trial conduct (page 10, lines 21-24 ; page 11, lines 1-3 ; page 12, lines 5-8) |
| Auditing | 23 | Frequency and procedures for auditing trial conduct, if any, and whether the process will be independent from investigators and the sponsor (Not applicable. This is the implementation of a process with data collection only at M12. There is therefore no need to have audit) |
| Ethics and dissemination | | |
| Research ethics approval | 24 | Plans for seeking research ethics committee/institutional review board (REC/IRB) approval (page 19, lines 19-24 ; page 20, lines 1-2) |
| Protocol amendments | 25 | Plans for communicating important protocol modifications (eg, changes to eligibility criteria, outcomes, analyses) to relevant parties (eg, investigators, REC/IRBs, trial participants, trial registries, journals, regulators) (page 13, lines 16-19) |
| Consent or assent | 26a | Who will obtain informed consent or assent from potential trial participants or authorised surrogates, and how (see Item 32) (page 6, lines 14-17) |
|  | 26b | Additional consent provisions for collection and use of participant data and biological specimens in ancillary studies, if applicable (page 19, lines 14-17) |
| Confidentiality | 27 | How personal information about potential and enrolled participants will be collected, shared, and maintained in order to protect confidentiality before, during, and after the trial (The information will be collected anonymously on a centralised eCRF) |
| Declaration of interests | 28 | Financial and other competing interests for principal investigators for the overall trial and each study site (page 20, lines 7-8) |
| Access to data | 29 | Statement of who will have access to the final trial dataset, and disclosure of contractual agreements that limit such access for investigators (page 19, lines 14-17) |
| Ancillary and post-trial care | 30 | Provisions, if any, for ancillary and post-trial care, and for compensation to those who suffer harm from trial participation (page 19, lines 6-12) |
| Dissemination policy | 31a | Plans for investigators and sponsor to communicate trial results to participants, healthcare professionals, the public, and other relevant groups (eg, via publication, reporting in results databases, or other data sharing arrangements), including any publication restrictions (page 13, line 24, page 14, lines 1-2 ; page 18, lines 1-5) |
|  | 31b | Authorship eligibility guidelines and any intended use of professional writers (page 13, lines 14-15 ; page 13, lines 22-23) |
|  | 31c | Plans, if any, for granting public access to the full protocol, participant-level dataset, and statistical code (page 13, line 24 ; page 14, lines 1-2) |
| Appendices |  |  |
| Informed consent materials | 32 | Model consent form and other related documentation given to participants and authorised surrogates (page 19, line 24 ; page 20, line 1) |
| Biological specimens | 33 | Plans for collection, laboratory evaluation, and storage of biological specimens for genetic or molecular analysis in the current trial and for future use in ancillary studies, if applicable (Not applicable : this study does not include collection of biological samples.) |

*It is strongly recommended that this checklist be read in conjunction with the SPIRIT 2013 Explanation & Elaboration for important clarification on the items. Amendments to the protocol should be tracked and dated. The SPIRIT checklist is copyrighted by the SPIRIT Group under the Creative Commons “[Attribution-NonCommercial-NoDerivs 3.0 Unported](http://www.creativecommons.org/licenses/by-nc-nd/3.0/)” license.
